# Deep Learning with Convolutional Neural Networks Applied to Electromyography Data: A Resource for the Classification of Movements for Prosthetic Hands

**DOI:** 10.3389/fnbot.2016.00009

**Published:** 2016-09-07

**Authors:** Manfredo Atzori, Matteo Cognolato, Henning Müller

**Affiliations:** Information Systems Institute, HES-SO Valais-Wallis, University of Applied Sciences Western SwitzerlandSierre, Switzerland

**Keywords:** electromyography, prosthetics, rehabilitation robotics, machine learning, deep learning, convolutional neural networks

## Abstract

Natural control methods based on surface electromyography (sEMG) and pattern recognition are promising for hand prosthetics. However, the control robustness offered by scientific research is still not sufficient for many real life applications, and commercial prostheses are capable of offering natural control for only a few movements. In recent years deep learning revolutionized several fields of machine learning, including computer vision and speech recognition. Our objective is to test its methods for natural control of robotic hands via sEMG using a large number of intact subjects and amputees. We tested convolutional networks for the classification of an average of 50 hand movements in 67 intact subjects and 11 transradial amputees. The simple architecture of the neural network allowed to make several tests in order to evaluate the effect of pre-processing, layer architecture, data augmentation and optimization. The classification results are compared with a set of classical classification methods applied on the same datasets. The classification accuracy obtained with convolutional neural networks using the proposed architecture is higher than the average results obtained with the classical classification methods, but lower than the results obtained with the best reference methods in our tests. The results show that convolutional neural networks with a very simple architecture can produce accurate results comparable to the average classical classification methods. They show that several factors (including pre-processing, the architecture of the net and the optimization parameters) can be fundamental for the analysis of sEMG data. Larger networks can achieve higher accuracy on computer vision and object recognition tasks. This fact suggests that it may be interesting to evaluate if larger networks can increase sEMG classification accuracy too.

## Introduction

Transradial amputees can be highly impaired, even if equipped with the most modern prostheses. The recent advances in deep learning and convolutional neural networks may contribute to help them recover some of their capabilities by bridging the gap between the prosthetics market (that requires fast and robust control methods) and recent scientific research results in rehabilitation robotics (that shows that dexterous and proportional control is possible).

Currently, the prosthetics market offers myoelectric prosthetic hands that are extremely advanced from a mechanical point of view and that can perform many different movements. However, the control methods are still in most cases rudimentary in order to guarantee 100% control robustness and sufficient control speed. Many myoelectric prosthetic hands are commercially available, however, few of them have the capability to reproduce many different movements. A selection of the most advanced prosthetic hands available in the market according to their movement capabilities currently include the following ones: (1) Vincent hand Evolution 2; (2) Steeper Bebionic v3; (3) Otto Bock Michelangelo; and (4) Touch Bionics i-limb Quantum (Atzori and Müller, 2015). Some of these prostheses are characterized by very high dexterity: they allow the movement of up to five different fingers independently. They allow the rotation of the thumb, to reproduce up to 36 different movements and the rotation of the wrist in near real time. In general, a commercial myoelectric prosthesis is opened or closed through the contraction of specific remnant muscles. While the mechanical characteristics of the mentioned prostheses are advanced, the control systems rely in most cases on specific movement triggers or sequential control strategies. Movement triggers link specific surface electromyography (sEMG) pulse sequences to specific movement of the prosthesis. Sequential control strategies allow to shift between a set of predefined movements through specific signals (e.g., through co-contraction, i.e., the simultaneous activation of two sEMG electrodes). Some of the considered prostheses include external sources of information in the form of active falling object prevention systems or via smartphones. Touch Bionics offers a selection of grasps according to objects located near the prosthesis (using Near-Field Communication, NFC) or according to action patterns (using accelerometer and gyroscope measurements). In the most advanced cases, pattern recognition is also used to control the prosthesis in combination with traditional methods. This solution has been proposed since 2013 by Coaptengineering and it was recently introduced by Touch Bionics to control wrist rotation. The mentioned control methods offer robust results, which are deemed to be one of the main needs in real use (Farina et al., 2014). However, the movement imagined to control the prosthesis is not natural, since it does not correspond to the movement that the amputee would have imagined to do, in order to control his real hand before the amputation. It also does not allow to control a large set of movements.

Proportional, natural and dexterous controls of robotic hand prostheses have been studied for a long time by scientific researchers. However, the current results are still not robust enough to be translated to real life use. Most of the methods rely on the use of sEMG and pattern recognition or proportional control algorithms. Pattern recognition algorithms are used to classify the movement that the subject aims to perform according to a label (Scheme and Englehart, 2011). The classification accuracy can be higher than 90–95% on less than 10 classes. However, average results are usually below 80–90% (Peerdeman et al., 2011). Simultaneous pattern recognition has been studied recently (Jiang et al., 2013; Ortiz-Catalan et al., 2013; Young et al., 2013). Proportional and simultaneous control of a large number of degrees of freedom of the prosthesis can allow achieving more natural and dexterous control using unsupervised or supervised methods (Fougner et al., 2012; Farina et al., 2014). Recently, semi-supervised methods and supervised methods were compared to evaluate the impact of precise kinematic estimations for accurately completing goal-directed tasks (Jiang et al., 2014).

Real time studies, allowing the user to adapt his response to the control software can provide a good representation of prosthesis usability (Hargrove et al., 2007; Scheme and Englehart, 2011). However, since these studies require the interaction of the user with the control system, they do not allow easy comparison with innovative analysis procedures. Another common problem in the field is that the studies are often highly specific and they are not directly comparable due to different acquisition setups, protocols and analysis pipelines. Moreover, often the datasets are not publicly available. The usefulness of benchmark databases has been demonstrated repeatedly in other fields, e.g., in the machine vision and image analysis communities (Müller et al., 2009; Everingham et al., 2010). Offline data analysis on public benchmark datasets allows the comparison of different methods and setups, accelerating the search and pushing forward progress in prosthetic control robustness. In 2014, the biggest publicly available benchmark database was released by the NinaPro project (Atzori et al., 2015). It consists of three datasets containing sEMG, accelerometer, and both hand kinematic and dynamic data recorded from 67 intact subjects and 11 amputees performing at least 50 hand movements.

Promising results have been obtained by invasive methods such as Peripheral Nerve Interfaces (Urbanchek et al., 2012), Cortical Interfaces (Chestek et al., 2011) or Targeted Muscle Reinnervation (TMR; Kuiken et al., 2009). The latter has shown very promising results, especially in transomeral or shoulder amputees (Atzori and Müller, 2015). TMR consists of the re-innervation of spare muscles of the amputee with the residual nerves of the amputated limb. However, the invasiveness of the procedure can strongly limit the application possibilities. A recent survey explored the interest of upper-limb amputees in four different techniques for prosthetic control: myoelectric, TMR, peripheral nerve interfaces, and cortical interfaces. Participants expressed the most interest in the myoelectric control, while the cortical interface elicited the lowest interest (Engdahl et al., 2015). This highlights that invasive techniques can be rejected by amputees.

Multimodal data acquisition has also been investigated. Computer vision has been combined with sEMG-based detection of movement intention to predetermine the type and size of the required grasp in relation to the object (Došen et al., 2010; Markovic et al., 2014). Accelerometers showed excellent capabilities to recognize hand movements using pattern recognition and regression methods, both alone and in combination with sEMG electrodes (Atzori et al., 2014c; Gijsberts et al., 2014; Krasoulis et al., 2015).

Nevertheless, despite several improvements on the market and scientific research, the robust natural control of dexterous prosthetic hands is still missing.

Deep learning and convolutional neural networks recently revolutionized several fields of machine learning, including speech recognition and computer vision. Thus, it seems reasonable to investigate its abilities in sEMG as well.

Despite it often being considered as a new and emerging field, the birth of deep learning can be set in the 1940’s. It passed through several stages and names over the years: born and known as *cybernetics*, it became popular as *connectionism* between the 1980’s and 1990’s, while since 2006 it started to be called with the current name (Goodfellow et al., 2016). In Goodfellow et al. (2016), the increasing dataset and model sizes are recognized as key points of the new success of this kind of approach. Thanks to the hardware and software advances it is now possible to use large networks trained with large datasets, allowing the exploitation of their capabilities.

Deep neural networks have been successful in several applications since the 1980’s. However, in the field of computer vision in 2012, deep learning approaches won one of the largest object recognition challenges (the ILSVRC) decreasing the previous top-5 error rate by more than 10% (Krizhevsky and Hinton, 2010; Goodfellow et al., 2016). Since then, only techniques based on convolutional neural networks have won this competition, leading to top-5 error rates lower than 5% (He et al., 2015; Goodfellow et al., 2016). Another remarkable result in computer vision was obtained in 2012, when human-level results were reached using multi-column deep neural networks on computer vision benchmarks (Cireşan et al., 2012). In the computer vision field, deep neural networks are also successfully applied in pedestrian detection (Sermanet et al., 2013) and traffic sign classification (Cireşan et al., 2012).

Since 2010, the application of deep learning techniques to speech recognition has allowed a quick and impressive reduction of error rate (Dahl et al., 2010; Deng et al., 2010b, 2013; Hinton et al., 2012; Goodfellow et al., 2016).

Deep learning methods are also successfully applied to applications requiring to process big amounts of data, such as drug discovery (Ramsundar et al., 2015), compound activity prediction (Dahl et al., 2014) and genomic information annotation (Chicco et al., 2014). Moreover, they have also improved the performance of reinforcement learning, where a machine or software agent is able to maximize its performance by itself performing trials and errors (Mnih et al., 2015; Goodfellow et al., 2016).

As reported, there are several and continuously increasing deep neural network applications. However, convolutional neural networks have been applied to sEMG hand movement recognition mainly in a single conference article. Park and Lee (2016) used a convolutional neural network model composed of an input layer, four convolutional layers, four subsampling layers, and two fully connected layers to improve inter-user variability in six hand movements via sEMG signals. The strategy adopted was to perform a first non-adaptation experiment, applying a trained model (or classifier) and a second experiment using a retrained model (or classifier) using few labeled data. The results show a better classification accuracy for the convolutional neural network compared to Support Vector Machines (SVM) in both experiments. The highest accuracy was reached using convolutional neural networks with the retrained network.

In this article, we apply convolutional neural networks to the classification of 50 hand movements in 67 intact subjects and 11 transradial hand amputees and we compare the results with those obtained with classical machine learning methods on three Ninapro datasets (Atzori et al., 2014b). The Ninapro database is particularly useful for this analysis since it provides publicly available data and reference classification performances with classical machine learning procedures.

## Materials and Methods

### Subjects

The data analyzed in this article are from the Ninapro database that includes electromyography data related to hand movements of 78 subjects (11 transradial amputees, 67 intact subjects) divided into three datasets. The Ninapro dataset 1 includes data acquisitions of 27 intact subjects (7 females, 20 males; 2 left handed, 25 right handed; age 28 ± 3.4 years). The second dataset includes data acquisitions of 40 intact subjects (12 females, 28 males; 6 left handed, 34 right handed; age 29.9 ± 3.9 years). The third dataset includes data acquisitions of 11 transradial amputees (11 males; 1 left handed, 10 right handed; age 42.36 ± 11.96 years). All participants signed an informed consent form. The experiment was approved by the Ethics Commission of the state of Valais (Switzerland), and it was conducted according to the principles expressed in the Declaration of Helsinki. More details about the subjects are reported in the official database description (Atzori et al., 2014b).

### Acquisition Setup and Protocol

#### Acquisition Setup

Several sensors were used to record hand kinematics, dynamics and correspondent muscular activity during the experiments. Hand kinematics were measured using a motion capture data glove with 22 sensors (CyberGlove II, CyberGlove Systems LLC). A 2-axis Kübler IS40 inclinometer (Fritz Kübler GmbH) was fixed onto the wrist of the subjects to measure the wrist orientation. Hand dynamics were measured using a Finger-Force Linear Sensor (FFLS; Kõiva et al., 2012).

Two types of double differential sEMG electrodes were used to record muscule activity. Dataset one was recorded using 10 OttoBock MyoBock 13E200-50 (Otto Bock HealthCare GmbH), providing an amplified, bandpass-filtered and Root Mean Square (RMS) rectified version of the raw sEMG signal at 100 Hz. The amplification of the electrodes was set to 5. These electrodes were fixed on the forearm using an elastic armband. Dataset 2 and 3 were recorded using 12 electrodes from a Delsys Trigno Wireless System, providing the raw sEMG signal at 2 kHz. These electrodes were fixed on the forearm using their standard adhesive bands and a hypoallergenic elastic latex-free band.

The sEMG electrodes are positioned in order to combine two methods that are common in the field, i.e., a dense sampling approach (Fukuda et al., 2003; Tenore et al., 2009; Li et al., 2010) and a precise anatomical positioning strategy (De Luca, 1997; Castellini et al., 2009). Eight electrodes were positioned around the forearm at the height of the radio humeral joint at a constant distance from each other; two electrodes were placed on the main activity spots of the flexor digitorum superficialis and of the extensor digitorum superficialis (Atzori et al., 2015; identified by palpation). In dataset 2 and 3, two electrodes were also placed on the main activity spots of the biceps brachii and of the triceps brachii (also in this case, identified by palpation). More details about the acquisition setup are reported in the official database descriptor (Atzori et al., 2014b).

#### Acquisition Protocol

Data acquisitions were performed with two types of exercises. In the first one, the subjects imitated several repetitions of hand movements that were shown on the screen of a laptop in the form of movies. In the second one, the subjects repeated nine force patterns by pressing with one or more hand digits on the FFLS. Several colored bars on the screen guided the subjects to increase the force exerted by each finger up to 80% of the maximal voluntary contraction force, and then back to 0%. Intact subjects were asked to imitate the movements with the right hand, while amputees were asked to imagine imitating the movements with the missing hand, as naturally as possible.

The entire acquisition protocol included several repetitions (10 repetitions for dataset 1, 6 repetitions for dataset 2 and 3) of 40 movements and nine force patterns that were selected from the hand taxonomy and robotics literature (Kamakura et al., 1980; Cutkosky, 1989; Edwards et al., 2002; Crawford et al., 2005; Sebelius et al., 2005; Kato et al., 2006; Feix et al., 2009) also in relationship to the activities of daily living (ADL). Movement repetitions lasted 5 s and were followed by 3 s of rest.

### Data Analysis

Data analysis aims at classifying data into an average of more than 50 classes (corresponding to hand movements) with convolutional neural networks and to compare the results with classical machine learning techniques.

#### Pre-Processing

For both classical and deep learning approaches, the following steps were executed. All the data streams were synchronized by super-sampling them to the highest sampling frequency (2 kHz or 100 Hz, depending on the used myoelectric electrodes) using linear interpolation. Since the movements performed by the subjects may not be perfectly synchronized with the stimuli proposed by the acquisition software due to human reaction times and experimental conditions, relabeling was performed offline with a generalized likelihood ratio algorithm (Kuzborskij et al., 2012). Since the Trigno electrodes are not shielded against power line interferences, their electromyography measurements were filtered from 50 Hz (and harmonics) power-line interference using a Hampel filter (Kuzborskij et al., 2012).

The test set consisted of approximately 1/3 of the movement repetitions (repetition 2, 5 and 7 in database 1; repetition 2 and 5 in database 2 and database 3). The training set consisted of the remaining repetitions. This approach is different from the leave-one-out approach used by Park and Lee (2016).

For classification using convolutional neural networks, after several preliminary tests (aimed to better understand the response of convolutional neural networks on sEMG), the Delsys trigno signals were made similar to Otto Bock’s by RMS rectification. Afterwards, the signal was subsampled at 200 Hz, in order to reduce computation time. Then, (both for the Delsys and the Otto Bock) the signals were low pass filtered at 1 Hz. Several normalization procedures were also tested during pre-processing in order to augment the performance of convolutional neural network classification, without leading to sensible improvement of the results.

#### Classification Using Convolutional Neural Networks

The convolutional neural network consisted of a modified version of a well known convolutional neural network (LeNet; LeCun et al., 1995), according to the implementation suggested for Cifar-10 in the package MatConvNet (Vedaldi and Lenc, 2015). The choice of a simple net, despite more complex recent ones being available, was performed in order to accelerate the training phase and to allow evaluating the effects of several pre-processing, architectural and optimization parameters according to the characteristics of the problem. While convolutional neural networks have been applied to many fields, including computer vision and speech recognition, their application to sEMG data is relatively novel (Park and Lee, 2016).

**Figure 1:**
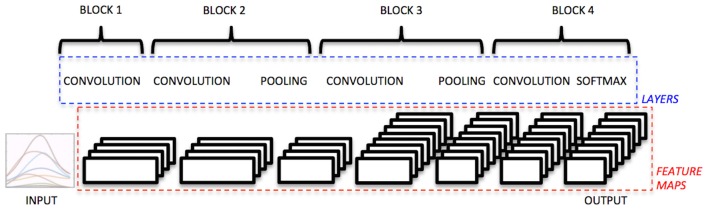
**Architecture of the convolutional neural network used on the sEMG signal**.

The architecture of the convolutional neural network (Figure 1) was structured as follows: the input data correspond to time windows of 150 ms, spanning all the electrode measurements available (10 for the Otto Bock, 12 for the Delsys). This choice corresponds well to what is usually done in the field, i.e., analyzing time windows aimed to allow control in real time (Englehart et al., 1999; Atzori et al., 2014b).

The first block of the net is composed of the following parts. First, it includes a convolutional layer composed of 32 filters. After several tests, including different shapes and sizes, the filters were defined as a row of the length of number of electrodes. Second, it includes a rectified linear unit as a non-linear activation function.

The second block of the net is composed of the following three parts. The first one is a convolutional layer with 32 filters of size 3 × 3. The second one is a non-linear activation function (rectified linear unit). The third one is a subsampling layer that performs an average pooling with filters of size 3 × 3.

The third block of the net is composed of the following three parts. The first one is a convolutional layer with 64 filters of size 5 × 5. The second one is a non linear activation function (rectified linear unit). The third one is a subsampling layer that performs an average pooling with filters of size 3 × 3.

The fourth block of the net is composed of the following two parts. The first is a convolutional layer with 64 filters of size 5 × 1 for the Otto Bock electrodes and size 9 × 1 for the Delsys electrodes. The second is a rectified linear unit.

The fifth block of the net is composed of the following two parts. The first one is a convolutional layer with filters of size 1 × 1. The second is a softmaxloss.

Several weight initializations were tested. Finally, the weights of the convolutional layers are initialized with random values in ranges determined in percentage according to the data range, in order to get reasonable training time and stability.

Hyper-parameters were identified via random search and manual hyper-parameter tuning (Bengio et al., 2015) on a validation set composed of two subjects randomly selected from dataset 1 and dataset 2. After several tests, the convolutional neural networks were trained using stochastic gradient descent with momentum 0.9, the learning rate was fixed at 0.001, the weight decay at 0.0005, the batch size was fixed at 256 and the number of epochs 30.

In order to increase accuracy, data augmentation was performed before training. In particular, data were doubled and white Gaussian noise was added to the new set with a signal to noise ratio equal to 25 of the measured power of the signal. Several data augmentation tests were made on the validation set, mainly changing the noise creation procedure. The selected method was chosen based on a balance between improvements and low computational time.

#### Reference Classical Classification

The procedure was based on the one described by Englehart et al. (Englehart and Hudgins, 2003; Gijsberts et al., 2014). It consisted of windowing at 200 ms, feature extraction and classification. Five signal features and three classification methods were considered, according to previous application to the Ninapro sEMG database and to sEMG in general (Englehart and Hudgins, 2003; Kuzborskij et al., 2012; Atzori et al., 2014b; Gijsberts et al., 2014). The selected signal features include: marginal Discrete Wavelet Transform (mDWT), Histogram (HIST), Waveform Length (WL), RMS and the normalized combination of all of them. The histogram (HIST) was divided into 20 bins along a 3σ threshold (Zardoshti-Kermani et al., 1995). The mDWT, was created with a db7 wavelet with three levels (Lucas et al., 2008). The used classifiers are well known, having previously been applied on sEMG in general and thoroughly described on the Ninapro data. They include: random Forests (Breiman, 2001), SVM (Cristianini and Shawe-Taylor, 2000) and k-Nearest Neighbors (Duda et al., 2001). The classification is performed on all the movements included in the database, including rest periods and the data are balanced according to the number of repetitions of movements. The reference classification procedure is described in detail in Atzori et al. (2014b).

## Results

Data analysis aimed at classifying an average of more than 50 hand movement meaning with an average chance level lower than 2%. As described in detail in the “Discussion” Section, the results can be compared only with sEMG classification problems targeting a similar number of classes (e.g., Atzori et al., 2014b, 2015). As previously shown (Atzori et al., 2016), results higher than 90% can be easily obtained with similar approaches by reducing the number of classes, even on amputees.

**Figure 2:**
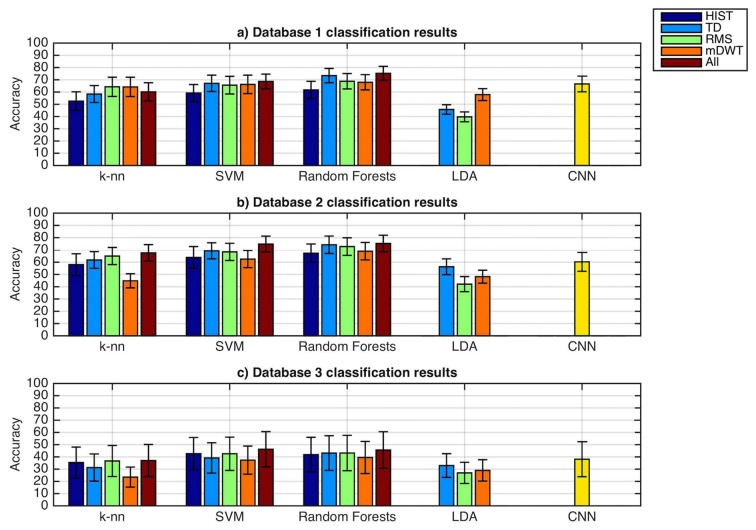
**Classification accuracy obtained with the classical classification techniques and the presented convolutional neural network.** The datasets include sEMG data with an average of more than 50 hand movements.

As represented in Figure 2, the classification accuracy obtained with convolutional neural networks using the simple architecture proposed is comparable with the average results obtained from classical classification techniques, but lower than the best results obtained with classical classification techniques.

The average classification accuracy obtained using the convolutional neural network on dataset 1 is 66.59 ± 6.40%. The average classification accuracy obtained using all the classical methods on this dataset is 62.06 ± 6.07%. The best classical classification method (Random Forests with all features) obtained an average classification accuracy of 75.32 ± 5.69%.

The average classification accuracy obtained using the convolutional neural network on dataset 2 is 60.27 ± 7.7%. The average classification accuracy obtained using all the classical methods on this dataset is 60.28 ± 6.51%. The best classical classification method (Random Forests with all features) obtained an average classification accuracy of 75.27% ± 7.89%.

For amputees (dataset 3), the average classification accuracy obtained using the convolutional neural network is 38.09 ± 14.29%. The average classification accuracy obtained using all the classical methods on this dataset is 38.82 ± 11.99%. The best classical classification method (SVM with all features) obtained an average classification accuracy of 46.27% ± 7.89%.

With convolutional neural networks (as well as with classical methods) the ratio between the accuracy and the chance level is in general higher than in previous results described in the literature for hand movement recognition in sEMG, e.g., 8.5 [10 movements, accuracy 84.4%, (Li et al., 2010)], 10.56 [12 movements, accuracy 87.8%, (Tenore et al., 2009)].

The average time required to train each convolutional neural network was 1 h and 42 min. The average time required to test the network was 21.5 s using an Nvidia Titan-x GPU. This leads to a time for the classification of each time window of less than 10^−3^ s.

Several network architectures, pre-processing parameters and hyperparameters were tested on a validation set, composed of three subjects randomly selected from dataset 1 and dataset 2. Depending on the case, the validation was made on all the movements available, or on a subset of eight movements. A summary of the results is reported in Table 1. The table reports the minimum Top-1 errors obtained for each parameter with the corresponding Top-5 error and epoch. Two different methods were tested: “time window normalization” (i.e., subtracting to each time window the mean and dividing it by the standard deviation) and “normalization based on training data” (i.e., subtracting to all the time windows the training data mean and dividing them by the training data standard deviation). The best results were obtained without any normalization procedure. Normalization procedures can affect the classification error up to 37%. Changing the learning rate can strongly change the minimum error for a fixed amount of epochs, while changes to the weight decay do not seem to affect the error substantially. Finally, data augmentation can reduce the classification error up to 4%, while also strongly reducing the number of epochs requested to reach it. A strong reduction of the error rate (48%) was obtained between the tests on normalization and the tests on the hyperparameters. This result was due to changes in the architecture of the net, in particular considering the first layer.

**Table 1:** **Tested pre-processing parameters and hyper-parameters**.

	Top-1 error	Top-5 error	Epoch
**1. Normalization (8 movements, different net)**
No Normalization	0.6	0.26	150
Time window normalization	0.97	0.88	200
Normalization based on training data	0.65	0.32	100
**2. Learning rate (8 movements)**
0.001	0.12	0.01	80
0.01	0.88	0.37	80
0.05	0.88	0.37	80
**3. Weight decay (8 movements)**
0.0001	0.12	0.01	80
0.0005	0.12	0.01	80
0.00005	0.12	0.01	80
**4. Data augmentation gaussian noise SNR ratio (all movements)**		
0	0.23	0.65	75
0.5	0.22	0.71	50
5	0.21	0.05	75
15	0.21	0.21	75
25	0.19	0.045	25
35	0.22	0.065	40
45	0.21	0.049	52
55	0.21	0.056	75

*The table reports the minimum Top-1 errors obtained for each parameter with the corresponding Top-5 error and epoch*.

In conclusion, the classification accuracy obtained with the proposed convolutional neural network is strongly influenced by several factors (including network architectures, pre-processing parameters and optimization parameters), it provides accuracy that is more precise than the average traditional methods in extremely little time, but it does not replicate the best classical classification methods for similar tasks.

## Discussion

During the last 5 years, deep learning and convolutional neural networks revolutionized several fields of machine learning, including speech recognition and computer vision. Thus, it seems reasonable to think that they may improve the analysis of sEMG and contribute to bridge the gap between prosthetics market (that requires fast and robust control methods) and recent scientific research results in rehabilitation robotics (that show that dexterous and proportional control is possible).

In this article, we introduce a baseline for the application of convolutional neural networks to the classification of hand movements by sEMG and we compare the results with a set of classical machine learning methods on a large set of movements and subjects (including also amputees).

The electromyography data of 67 intact subjects and 11 hand amputees performing an average of more than 50 hand movements were analyzed. The data are publicly available on the Ninapro database (Atzori et al., 2014b) and they are divided into three datasets including 27, 40 and 11 subjects respectively.

The results show that convolutional neural networks with a very simple architecture are comparable to the average classical machine learning classification methods and they show that several factors (including pre-processing, the architecture of the net and the optimization parameters) are fundamental for the analysis of sEMG data. Convolutional neural network results obtained with the very simple architecture described in this article are not worse than the average of classical methods, thus we believe that they are a good avenue to explore.

The classification accuracy obtained with convolutional neural networks using the proposed architecture is 66.59 ± 6.4% on dataset 1, 60.27 ± 7.7% on dataset 2 and 38.09 ± 14.29% on amputees (dataset 3). The average results are comparable to the average results obtained with the reference classical classification, but lower than the results obtained with the best classical classification techniques. The results described in this article represent one of the first attempts to train a simple convolutional neural network on sEMG data. The literature for computer vision and object recognition showed that larger networks can achieve higher accuracy on complex tasks (Bengio et al., 2015). Thus, it may be interesting to evaluate if larger networks can improve sEMG classification too.

Regarding the overall accuracy (obtained both with convolutional neural networks and classical methods), it is fundamental to note that the results should be compared only with analyses considering a similar number of classes, i.e., approximately 50. The chance level varies with the number of classes. Therefore, considering a dataset (with a specific number of samples), a feature set and a classifier, classification accuracy is expected to decrease when the number of classes increases (Deng et al., 2010a). Thus, it is fundamental to compare accuracy only when the number of classes is comparable. It is common to see in the literature movement classification accuracy of up to 90%–95% (Castellini and van der Smagt, 2009; Tenore et al., 2009; Li et al., 2010; Peerdeman et al., 2011). However, most of these studies consider between 4 and 12 movements, with chance level between 25% and 8.33%, while the chance level of this study is inferior to 2%. Thus, a comparison of the accuracy would not be reasonable and justified by statistics. As previously shown, results over 90% of accuracy can be obtained reducing the number of classified movements to approximately 10 for amputees, even starting from lower classification accuracies (Atzori et al., 2014a; Atzori et al., 2016). Moreover, classification accuracy can change strongly depending on several other parameters [including e.g., class balance and for amputees, several clinical parameters including forearm percentage, phantom limb sensation and years from the amputation (Atzori et al., 2016)]. Therefore, comparisons in this field must not be made lightly.

Pre-processing, net architecture and the optimization parameters seem to be fundamental for the analysis of sEMG data with convolutional neural networks, since they can strongly change the final classification accuracy in the validation set, and time to converge. The factors that influenced the most the results were the shape of the first layer of the network, the initial weights of the layers, data augmentation procedures and the learning rate.

The net architecture that was chosen is extremely simple. This choice was made on purpose, in order to make it easier to evaluate the effect of changes in the pre-processing, in the architecture of the net and in the optimization parameters. However, more complex net architectures do exist and can be trained on sEMG data, thus probably leading to higher accuracies. This fact is extremely promising for the future of sEMG data analysis and rehabilitation robotics, and may lead to increased dexterous control of robustness, thus contributing to bridge the gap between the prosthetics market and scientific research.

In conclusion, the baseline results that have been presented in this article show that convolutional neural networks with very simple architecture can produce accurate results comparable to the average classical classification methods, and they suggest that further studies may lead to improve the overall field of sEMG controlled dexterous hand prosthetics.

## Author Contributions

MA analyzed the data and wrote the manuscript. MC analyzed the state of the art and wrote the manuscript. HM supervised the analysis and wrote the manuscript.

## Funding

This work is partially supported by the Swiss National Science Foundation Sinergia project # 160837 Megane Pro.

## Conflict of Interest Statement

The authors declare that the research was conducted in the absence of any commercial or financial relationships that could be construed as a potential conflict of interest.
